# Factors influencing DVT formation in sepsis

**DOI:** 10.1186/s12959-024-00582-y

**Published:** 2024-01-16

**Authors:** Lu Wang, Xudong Ma, Yujie Chen, Sifa Gao, Wei Pan, Jieqing Chen, Longxiang Su, Huaiwu He, Yun Long, Chang Yin, Xiang Zhou, On Behalf of China National Critical Care Quality Control Centre Group (China-NCCQC)

**Affiliations:** 1 Department of Critical Care Medicine, State Key Laboratory of Complex Severe and Rare Diseases, Peking Union Medical College, Peking Union Medical College Hospital, Chinese Academy of Medical Sciences, Beijing, 100730 China; 2 Department of Medical Administration, National Health Commission of the People’s Republic of China, Beijing, 100044 China; 3 Information Center Department, Department of Information Management, Peking Union Medical College, Peking Union Medical College Hospital, Chinese Academy of Medical Sciences, Beijing, 100730 China; 4 National Institute of Hospital Administration, Beijing, 100730 China

**Keywords:** DVT, Sepsis, SOFA, Coagulation, Respiration, Liver

## Abstract

**Introduction:**

Sepsis is a global public health burden. Deep vein thrombosis (DVT) is the third most common cause of death from cardiovascular disease after heart attacks and strokes. We designed this experiment to investigate the factors influencing DVT formation in patients with sepsis.

**Methods:**

In this survey, 918 septic patients admitted to Peking Union Medical College Hospital, who underwent DVT screening were enrolled. The data were collected from June 8, 2013 to October 12, 2022. The differences between septic patients with and without DVT were studied from following aspects: basic information, comorbidities, inflammatory cytokines, albumin, source of infection, sequential organ failure assessment (SOFA) score, coagulation and prognosis.

**Main results:**

In this study, the prevalence of DVT in patients with sepsis was 0.23. Elderly patients with sepsis were prone to DVT (*p* value < 0.001). In terms of comorbidities, septic patients with hypertension and atrial fibrillation were prone to DVT (*p* value 0.045 and 0.048). Inflammatory cytokines, such as procalcitonin (PCT), C-reactive protein (CRP), interleukin (IL)-6, IL-8, IL-10, tumor necrosis factor (TNF)-α, had no significant correlation with DVT in patients with sepsis (*p* value 0.364, 0.882, 0.912, 0.789, 0.245, and 0.780). Levels of serum albumin correlated with DVT in patients with sepsis (*p* value 0.003). The SOFA total score had no relationship with DVT formation (*p* value 0.254). Coagulation and respiration function were negatively correlated with DVT (*p* value 0.018). Liver function was positively correlated with DVT (*p* value 0.020). Patients in the DVT group had longer duration of mechanical ventilation and longer intensive care unit (ICU) stays (*p* value < 0.001 and 0.006). There was no significant difference in survival in septic patients with and without DVT (*p* value 0.868).

**Conclusions:**

The SOFA total score had no relationship with DVT formation. The function of each organ had different effects on DVT formation. Better coagulation and respiration function, easier DVT formation. Poorer liver function, easier DVT formation. DVT was associated with longer duration of mechanical ventilation and longer ICU stays.

## Introduction

Deep vein thrombosis (DVT) is the third most common cause of death from cardiovascular disease after heart attacks and strokes [1]. Sepsis is life-threatening organ dysfunction caused by a dysregulated host response to infection [2]. Sepsis affects millions of people worldwide and is one of the largest causes of death worldwide [3]. To reduce mortality from sepsis, the Surviving Sepsis Campaign has released five sets of guidelines over the last 20 years, with the most recent being published in 2021 [4]. The Virchow triad of DVT formation (endothelial lesions, hypercoagulability status, and venous stasis) is prevalent in patients with septic shock. However, DVT in sepsis is poorly studied and mostly remains theoretical [5–8]. Common risk factors for DVT include old age, obesity, malignancy, myocardial infarction, heart failure, vasculitis, systemic lupus erythematosus, nephrotic syndrome, hypertension, diabetes mellitus, polycythemia vera, and thrombocytosis. There is little literature on the influence of these factors on DVT formation in patients with sepsis [9, 10], so we designed this experiment to investigate the prevalence of DVT in patients with sepsis and the influence of the aforementioned high-risk factors on DVT formation.

## Methods

### Study Design

**Fig. 1:**
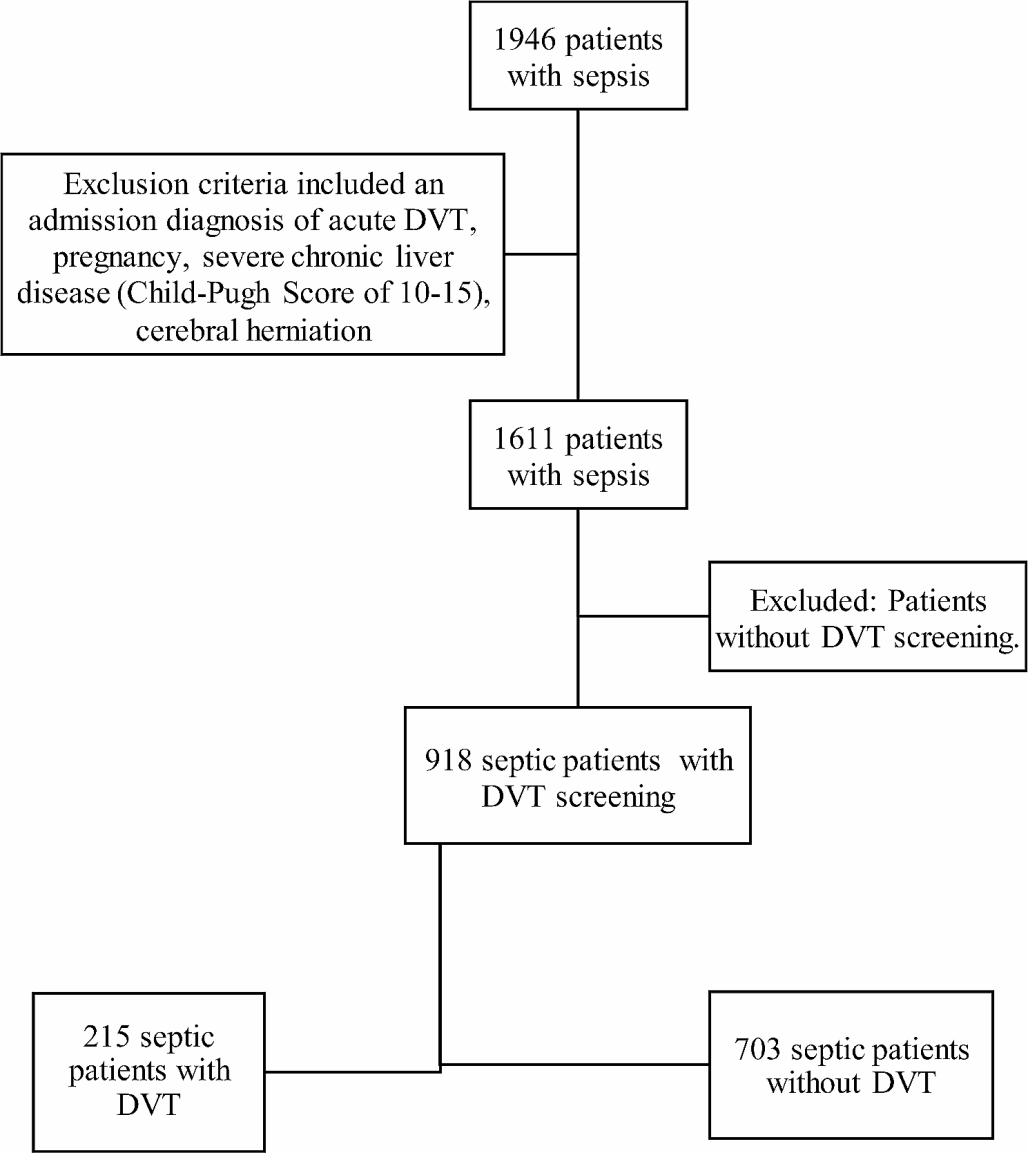
DVT screening process. DVT = deep vein thrombosis

In this survey, 918 patients with sepsis admitted to Peking Union Medical College Hospital were enrolled. Of these sepsis patients, 215 had DVT, which occurred in 23.42% of cases. The data were collected from June 8, 2013 to October 12, 2022. Sepsis was diagnosed on the basis of the third international consensus definitions for sepsis and septic shock [2]. Exclusion criteria included an admission diagnosis of acute DVT, without screening for DVT, pregnancy, severe chronic liver disease (Child-Pugh Score of 10–15), and cerebral herniation (Fig. 1).

According to the management requirements of Peking Union Medical College Hospital, all patients in this study received DVT prophylaxis. Standardized protocols allowed for the use of either low-dose unfractionated heparin or low-molecular-weight heparin as appropriate. If patients had a contraindication to pharmacologic-based DVT prophylaxis (e.g., active bleeding or high risk of bleeding), intermittent pneumatic compressions or graduated compression stockings were used.

The authors are accountable for all aspects of the work in ensuring that questions related to the accuracy or integrity of any part of the work are appropriately investigated and resolved. The datasets supporting the conclusions of this article are included within the article.

### Variables and measurements

Patients included in this study had completed at least one DVT screening during their ICU period. Lack of venous compressibility with the ultrasound transducer held in a transverse position to the vein was interpreted as a positive study of DVT. All compression ultrasonography were interpreted by board-certified sonographer blinded to the patient’s clinical history. Ultrasonography was coded as negative (DVT absent) if all imaged deep vein segments were fully compressible or as positive (DVT present) if a noncompressible segment was identified. Decisions on VTE treatment were left to the discretion of the patient’s primary team.

We studied the differences between septic patients with and without DVT from the following aspects: basic information, comorbidities, source of infection, inflammatory cytokines at onset of sepsis, albumin at onset of sepsis, sequential organ failure assessment (SOFA) score at onset of sepsis, coagulation at onset of sepsis and prognosis. Basic information included gender, age, height, weight, and body mass index (BMI). Comorbidities included diabetes, hypertension, coronary heart disease (CHD), chronic obstructive pulmonary disease (COPD), chronic kidney disease (CKD), immune diseases, malignancy, atrial fibrillation, and stroke. Source of infection included respiratory, abdominal, bloodstream, urinary tract, neurological, and others. Inflammatory cytokines at onset of sepsis included procalcitonin (PCT), C-reactive protein (CRP), interleukin (IL) -6, IL-8, IL-10, and tumor necrosis factor (TNF) -α. Coagulation at onset of sepsis included prothrombin time (PT), activated partial thromboplastin time (APTT), fibrinogen, D-dimer, and platelet. Prognosis included duration of mechanical ventilation, ICU stays, and the survival rate.

### Ethical considerations

The current study was reported in accordance with the Strengthening the Reporting of Observational Studies in Epidemiology Guidelines. This study was conducted in accordance with the Declaration of Helsinki (as revised in 2013). The trial protocol was approved by the Central Institutional Review Board at Peking Union Medical College Hospital (NO. SK1828), and individual consent for this analysis was waived. There was no identifying or protected health information included in the analyzed dataset.

### Data analysis

All statistical analyses were performed in SAS 9.4 (SAS Institute Inc., Cary, NC, USA). Continuous variables are expressed as media (P25, P75). The pairwise comparison was conducted by using the t test of two independent samples. All statistical tests were two-tailed, and *p* < 0.05 was considered to be statistically significant.

## Results

**Fig. 2:**
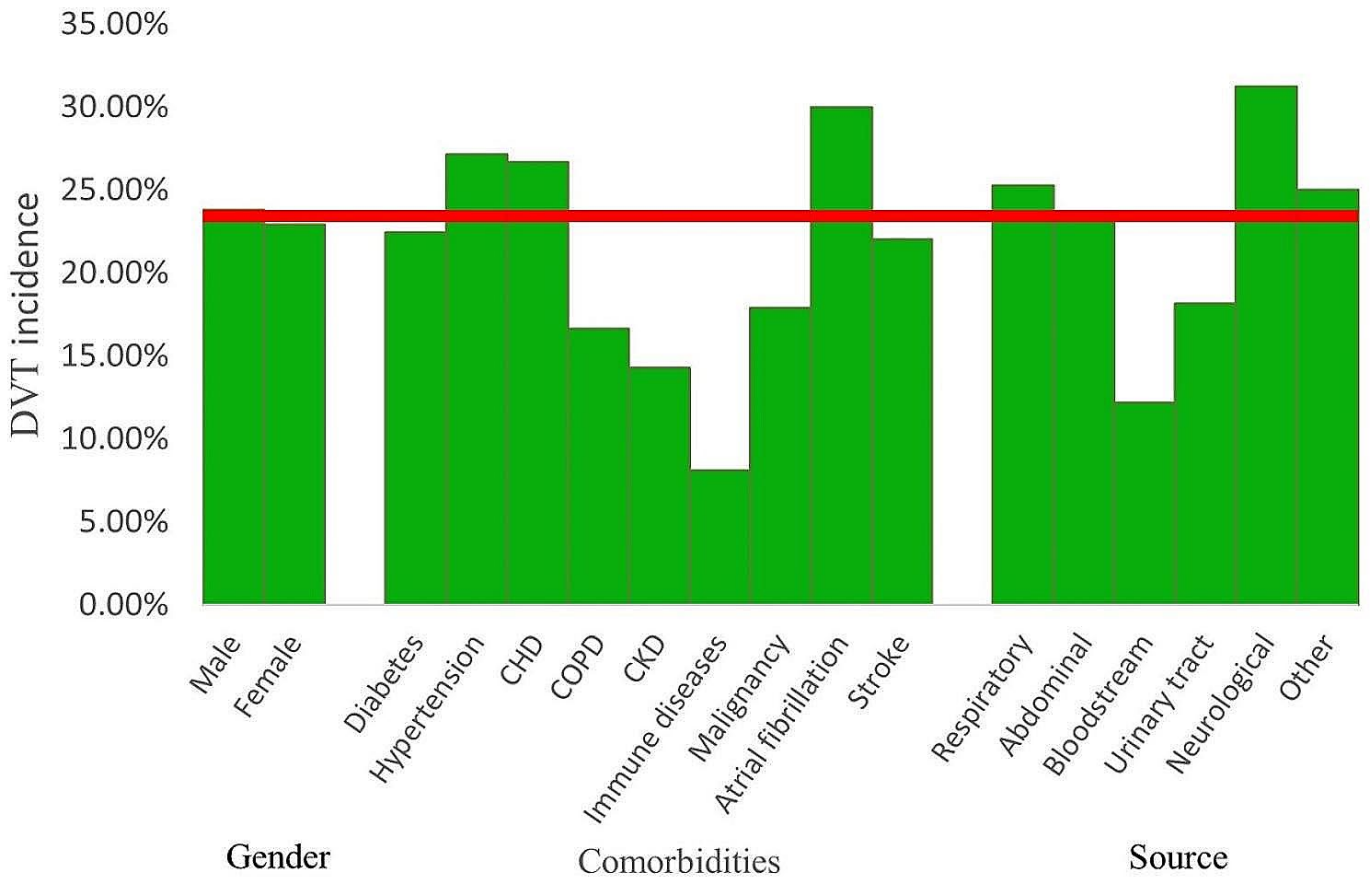
Incidence of DVT. DVT = deep vein thrombosis, CHD = coronary heart disease, COPD = chronic obstructive pulmonary disease, CKD = chronic kidney disease

In this study, the prevalence of DVT in patients with sepsis was 23.42%. In terms of comorbidities, patients with atrial fibrillation had the highest incidence of DVT, reaching 30.00%. In terms of source, patients with neurological infection had the highest incidence of DVT, reaching 31.25%, while patients with bloodstream infection had the lowest incidence of DVT, at 12.20% (Fig. 2).

Elderly patients with sepsis were prone to DVT (*p* value < 0.001). In terms of comorbidities, septic patients with hypertension and atrial fibrillation were prone to DVT (*p* value 0.045 and 0.048). However, septic patients with chronic kidney disease (CKD), immunological diseases, and malignancy were not prone to DVT (*p* value 0.008, < 0.001 and 0.024) (Table 1).

**Table 1:** Basic information and comorbidities

	Total	DVT	No DVT	OR	p
Basic information					
Female (n)	401	92	309	1.05 [0.77;1.43]	0.824
Age (y)	63.0 [51.0–73.0]	67.0 [58.0–76.0]	61.0 [48.0–72.0]	0.98 [0.97;0.98]	< 0.001
Height (cm)	168 [160–174]	170 [160–175]	167 [160–173]	0.99 [0.96;1.02]	0.365
Weight (kg)	65.0 [56.5–75.0]	66.0 [60.0-76.5]	65.0 [55.0–73.0]	0.99 [0.98;1.01]	0.202
BMI (kg/m^2^)	23.4 [20.8–26.1]	24.2 [21.0-26.1]	23.1 [20.3–26.1]	0.99 [0.95;1.04]	0.332
Comorbidities					
Diabetes (n)	227	51	176	1.07 [0.75;1.54]	0.764
Hypertension (n)	346	94	252	0.72 [0.53;0.98]	0.045
CHD (n)	161	43	118	0.81 [0.55;1.20]	0.326
COPD (n)	48	8	40	1.54 [0.74;3.61]	0.337
CKD (n)	140	20	120	1.99 [1.23;3.38]	0.008
Immune diseases (n)	173	14	159	4.15 [2.43;7.68]	< 0.001
Malignancy (n)	240	43	197	1.55 [1.08;2.28]	0.024
Atrial fibrillation (n)	150	45	105	0.66 [0.45;0.98]	0.048
Stroke (n)	245	54	191	1.11 [0.79;1.59]	0.612

Deep vein thrombosis = DVT, body mass index = BMI, coronary heart disease = CHD, chronic obstructive pulmonary disease = COPD, chronic kidney disease = CKD

In terms of source, patients with bloodstream infections were less likely to develop DVT (*p* value 0.017) (Table 2).

**Table 2:** Source of infection, inflammatory cytokines, and albumin

	Total	DVT	No DVT	OR	p
Source					
Respiratory (n)	494	125	369	0.80 [0.58;1.08]	0.169
Abdominal (n)	234	55	179	0.99 [0.70;1.42]	1.000
Bloodstream (n)	82	10	72	2.31 [1.22;4.86]	0.017
Urinary tract (n)	60	13	47	1.10 [0.60;2.17]	0.862
Neurological (n)	60	13	47	1.10 [0.60;2.17]	0.862
Other (n)	48	12	36	0.91 [0.47;1.85]	0.928
Inflammatory cytokines					
PCT (ng/L)	1.80 [0.42–7.28]	1.94 [0.47–8.38]	1.70 [0.40–7.04]	1.00 [0.99;1.00]	0.364
CRP (mg/L)	61.0 [24.0–98.5]	51.0 [28.0–94.0]	67.0 [23.0–102]	1.00 [0.99;1.01]	0.882
IL-6 (pg/mL)	52.4 [15.6–114]	52.6 [15.8–105]	52.2 [15.6–116]	1.00 [1.00;1.00]	0.912
IL-8 (pg/mL)	96.0 [50.5–208]	92.0 [62.0-156]	97.0 [45.5–245]	1.00 [1.00;1.00]	0.789
IL-10 (pg/mL)	8.75 [5.05–17.8]	8.30 [5.00–14.5]	9.10 [5.30–22.1]	1.00 [1.00;1.00]	0.245
TNF-α (pg/mL)	19.5 [12.9–31.6]	19.5 [13.6–25.9]	19.5 [12.0-33.2]	1.02 [1.00;1.03]	0.780
Albumin (g/L)	29.0 [25.0–32.0]	30.0 [26.0–34.0]	28.0 [24.8–32.0]	0.98 [0.96;1.00]	0.003

Deep vein thrombosis = DVT, procalcitonin = PCT, C-reactive protein = CRP, interleukin = IL, tumor necrosis factor = TNF

Inflammatory cytokines, such as procalcitonin (PCT), C-reactive protein (CRP), interleukin (IL)-6, IL-8, IL-10, and tumor necrosis factor (TNF)-α, had no significant correlation with DVT in patients with sepsis (*p* value 0.364, 0.882, 0.912, 0.789, 0.245, and 0.780) (Table 2).

The levels of serum albumin correlated with DVT in patients with sepsis (*p* value 0.003) (Table 2).

The SOFA total score had no relationship with DVT formation (*p* value 0.254). In the initial analysis, we found that only coagulation scores were negatively correlated with DVT (*p* value 0.007). However, when we switched to the raw data, we found that both respiration and liver were associated with DVT formation. Respiratory function was negatively correlated with DVT (*p* value 0.018). Liver function was positively correlated with DVT (*p* value 0.020) (Table 3).

**Table 3:** SOFA score and coagulation

	Total	DVT	No DVT	OR	p
SOFA score	12.0 [10.0–16.0]	13.0 [10.0–16.0]	12.0 [10.0–15.0]	0.98 [0.94;1.02]	0.254
Respiration score	2.00 [2.00–3.00]	2.00 [2.00–3.00]	2.00 [2.00–3.00]	0.87 [0.73;1.05]	0.080
Coagulation score	2.00 [0.00–3.00]	1.00 [0.00–2.00]	2.00 [1.00–3.00]	1.21 [1.06;1.39]	0.007
Liver score	0.00 [0.00–2.00]	1.00 [0.00–2.00]	0.00 [0.00–2.00]	0.91 [0.78;1.06]	0.102
Cardiovascular score	4.00 [1.00–4.00]	4.00 [2.00–4.00]	4.00 [1.00–4.00]	0.92 [0.83;1.02]	0.137
CNS score	4.00 [4.00–4.00]	4.00 [4.00–4.00]	4.00 [4.00–4.00]	0.90 [0.73;1.12]	0.205
Renal score	1.00 [0.00–3.00]	1.00 [0.00–3.00]	0.50 [0.00–3.00]	0.95 [0.86;1.05]	0.286
PaO2 / FiO2 (mmHg)	170 [102–254]	183 [115–272]	166 [99.8–243]	1.00 [1.00;1.00]	0.018
Platelet (10^9/L)	112 [60.0–174]	118 [80.0–186]	108 [53.0–168]	1.00 [1.00;1.00]	0.008
TBIL (umol/L)	19.8 [10.6–36.5]	21.9 [12.7–39.5]	18.2 [9.78–35.7]	1.00 [1.00;1.00]	0.020
NE (ug/kg/min)	0.11 [0.00–0.19]	0.11 [0.00–0.20]	0.11 [0.00–0.18]	1.06 [0.64;1.75]	0.308
Creatinine (umol/L)	83.0 [58.0–138]	86.0 [56.0–132]	82.0 [59.0–141]	1.00 [1.00;1.00]	0.887
Coagulation					
PT (s)	14.5 [13.1–16.5]	14.8 [13.4–16.6]	14.4 [13.1–16.4]	0.99 [0.96;1.02]	0.076
APTT (s)	36.3 [30.2–45.4]	35.4 [30.4–43.5]	36.6 [30.1–46.2]	1.01 [1.00;1.02]	0.403
Fibrinogen (g/L)	3.59 [2.34–4.69]	3.82 [2.70–5.03]	3.53 [2.30–4.61]	0.93 [0.86;1.01]	0.033
D-dimer (mg/L)	5.02 [2.99–10.7]	6.03 [3.60–10.6]	4.74 [2.86–10.7]	1.00 [0.99;1.02]	0.097
Platelet (10^9/L)	112 [60.0–174]	118 [80.0–186]	108 [53.0–168]	1.00 [1.00;1.00]	0.008

Sequential organ failure assessment = SOFA, deep vein thrombosis = DVT, central nervous system = CNS total bilirubin = TBIL, norepinephrine = NE, prothrombin time = PT, activated partial thromboplastin time = APTT

Among the commonly used coagulation indicators, prothrombin time (PT) and activated partial thromboplastin time (APTT) were not significantly related to DVT formation (*p* value 0.076 and 0.403). Fibrinogen levels were positively correlated with DVT formation (*p* value 0.033). Although the difference was not statistically significant (*p* value 0.097), patients in the DVT group had higher levels of D-dimer than those in the non-DVT group (Table 3).

Patients in the DVT group had a longer duration of mechanical ventilation and longer intensive care unit (ICU) stays (*p* value < 0.001 and 0.006). There was no significant difference in survival in septic patients with and without DVT (*p* value 0.868) (Table 4).

**Table 4:** Prognosis

	Total	DVT	No DVT	OR	p
Prognosis					
Ventilator (h)	85 [5.00–273]	135 [27.0–354]	70 [0.00–240]	1.00 [1.00;1.00]	< 0.001
ICU stay (h)	188 [73.0–411]	281 [82.6–540]	167 [71.9–380]	1.00 [1.00;1.00]	0.006
Survivor (n)	634 (69.1%)	147 (68.4%)	487 (69.3%)	1.04 [0.75;1.45]	0.868

Deep vein thrombosis = DVT, intensive care unit = ICU

## Discussion

Although DVT prophylaxis was performed, the incidence of DVT in patients with sepsis was still as high as 23.42%. Given the severity of the consequences [11, 12] and the ease of screening [13, 14], there is reason to believe that DVT screening in patients with sepsis should be popularized. In terms of comorbidities, the incidence of DVT was highest in septic patients with atrial fibrillation. In terms of source, patients with neurological infections had the highest incidence of DVT. These results are consistent with those of other relevant reports [15–18], and DVT prevention and treatment in septic patients with atrial fibrillation and neurological infection should be improved.

In patients with sepsis, elderly age [19], hypertension [20], and atrial fibrillation [21] are factors that predispose patients to DVT, which is consistent with other reports. Particular attention should be given to the prevention and treatment of DVT in septic patients who have the above risk factors.

Septic patients with CKD, immunological diseases, and malignancy were not prone to DVT in this study. These factors are strongly associated with DVT [22, 23], and the current seemingly counterintuitive results may be because patients with these risk factors tend to have stronger basic anticoagulation therapy [24–26], leading to bias in the results.

In terms of source, patients with bloodstream infections were less likely to develop DVT. This may be because sources such as catheters are easier to remove and bloodstream infections tend to have a shorter duration of treatment than other infections [27, 28].

Contrary to most current theories, inflammatory cytokines had no significant correlation with DVT in patients with sepsis in this study. We speculate that these results may be due to inflammatory factors lead to vascular endothelial damage [29, 30], and these inflammatory factors leading to a hyperdynamic phase with high cardiac output [31, 32]. This leads to accelerated venous return, and the two above effects cancel each other out.

Albumin is the most commonly used colloidal fluid in the treatment of sepsis [33], and its use in sepsis is still highly controversial [34, 35]. In this study, the levels of serum albumin correlate with DVT in patients with sepsis. The cause of this results may be elevated serum albumin levels leading to hypercoagulation. Albumin plays a critical role in restoring endothelial basement membrane integrity, and optimizing hemostasis in hemorrhagic shock [36]. This result suggests that changes in coagulation status should be concerned when using albumin in clinical practice.

SOFA is based on six different scores, one for each of the respiratory, cardiovascular, hepatic, coagulation, renal and neurological systems, and each is scored from 0 to 4. SOFA is the most important scoring system for diagnosing sepsis and assessing the severity of its condition [33]. This study investigated the relationship between SOFA scores and DVT formation. The SOFA total score was no correlated with DVT formation. Coagulation and respiration function was negatively correlated with DVT. Liver function was positively correlated with DVT. The combination of individual organs led to SOFA scores independent of DVT formation. Among the commonly used coagulation indicators, PT and APTT were not significantly related to DVT formation. At present, the prevention and treatment of DVT focuses on anticoagulation [37, 38], while anti- platelet research is less common [39]. In our study, platelet count was positively correlated with DVT formation, while anticoagulation was not significantly associated with DVT formation. These results suggest that in the prevention and treatment of DVT, more attention should be given to the adjustment of platelet function in addition to anticoagulation. Unexpectedly, the analysis results of respiration function showed that the degree of hypoxia was inversely correlated with DVT, and patients with severe hypoxia were less prone to DVT. We speculate that these results may be due to hypoxia inducing the release of inflammatory factors, leading to a hyperdynamic phase and accelerated venous return [31, 32]. In the initial analysis of this study, we found that only coagulation scores were correlated. However, when we replaced the original data, we found that both the respiratory system and liver were associated with DVT formation. This result suggests that we should use raw data for relevant research.

Patients in the DVT group exhibited a longer duration of mechanical ventilation and longer ICU stays. There was no significant difference in survival in septic patients with and without DVT. Matthew T. Rondina’s research shown that patients with sepsis with clinically significant venous thromboembolism had a significantly longer ICU stays compared with patients without venous thromboembolism. All-cause, 28-day mortality was numerically higher in patients with clinically significant venous thromboembolism but did not reach statistical significance [10]. Our study presents approximate results with Matthew T. Rondina’s research. These above results suggest that DVT prevention and treatment may not affect the survival of patients with sepsis, but may shorten the duration of their mechanical ventilation and hospital stays [40, 41].

There are some limitations to this study. First, since the present study was retrospective, all patients were not regularly screened for DVT. Patients may have DVT, but it is not detected in time. Second, this is a cross-sectional study and no dynamic monitoring data are available. Third, this study is retrospective and has many confounding factors. Thus, prospective studies are needed to further confirm the relevant conclusions.

## Conclusion

The SOFA total score did not demonstrate a relationship with DVT formation. The function of each organ had different effects on DVT formation. Better coagulation and respiration function resulted in easier DVT formation. Poorer liver function was associated with easier DVT formation. DVT was associated with a longer duration of mechanical ventilation and longer ICU stays.

## Data Availability

No datasets were generated or analysed during the current study.

## Abbreviations

DVT: Deep vein thrombosis
ICU: intensive care unit
BMI: body mass index
SOFA: sequential organ failure assessment
CHD: coronary heart disease
COPD: chronic obstructive pulmonary disease
CKD: chronic kidney disease
PCT: procalcitonin
CRP: C-reactive protein
IL: interleukin
TNF: tumor necrosis factor
CNS: central nervous system
TBIL: total bilirubin
NE: norepinephrine
PT: prothrombin time
APTT: activated partial thromboplastin time
PT: prothrombin time
